# Will Sirtuins Be Promising Therapeutic Targets for TBI and Associated Neurodegenerative Diseases?

**DOI:** 10.3389/fnins.2020.00791

**Published:** 2020-07-31

**Authors:** Qianjie Yang, Yunxiang Zhou, Yuting Sun, Yi Luo, Ye Shen, Anwen Shao

**Affiliations:** ^1^Department of Ophthalmology, The First Affiliated Hospital, Zhejiang University School of Medicine, Hangzhou, China; ^2^Department of Surgical Oncology, The Second Affiliated Hospital, School of Medicine, Zhejiang University, Hangzhou, China; ^3^The Second Affiliated Hospital, Zhejiang University School of Medicine, Hangzhou, China; ^4^Department of Neurosurgery, The Second Affiliated Hospital, School of Medicine, Zhejiang University, Hangzhou, China

**Keywords:** sirtuin, traumatic brain injury, neurodegenerative disease, neuroinflammation, excitotoxicity, oxidative stress

## Abstract

Traumatic brain injury (TBI), a leading cause of morbidity worldwide, induces mechanical, persistent structural, and metabolic abnormalities in neurons and other brain-resident cells. The key pathological features of TBI include neuroinflammation, oxidative stress, excitotoxicity, and mitochondrial dysfunction. These pathological processes persist for a period of time after TBIs. Sirtuins are evolutionarily conserved nicotinamide-adenine dinucleotide (NAD+)-dependent deacetylases and mono-ADP-ribosyl transferases. The mammalian sirtuin family has seven members, referred to as Sirtuin (SIRT) 1–7. Accumulating evidence suggests that SIRT1 and SIRT3 play a neuroprotective role in TBI. Although the evidence is scant, considering the involvement of SIRT2, 4–7 in other brain injury models, they may also intervene in similar pathophysiology in TBI. Neurodegenerative diseases are generally accepted sequelae of TBI. It was found that TBI and neurodegenerative diseases have many similarities and overlaps in pathological features. Besides, sirtuins play some unique roles in some neurodegenerative diseases. Therefore, we propose that sirtuins might be a promising therapeutic target for both TBI and associated neurodegenerative diseases. In this paper, we review the neuroprotective effects of sirtuins on TBI as well as related neurodegeneration and discuss the therapeutic potential of sirtuin modulators.

## Introduction

Traumatic brain injury (TBI) is a leading cause of morbidity worldwide. It is associated with long-term disability and significant healthcare expenditures and has become a priority for public health policy (Quaglio et al., 2017). Although most of the neurological injuries are limited and temporary (Cornelius et al., 2013), TBI still has multiple sequelae including sleep disturbance (Barshikar and Bell, 2017), hypopituitarism (Klose and Feldt-Rasmussen, 2018), seizures, epilepsy (Zimmermann et al., 2017), and neurodegenerative diseases (Gardner and Yaffe, 2015). Indeed, TBI is widely recognized as a risk factor for neurodegenerative diseases (Goldstein et al., 2012; Gardner and Yaffe, 2015; Daglas and Adlard, 2018; Gardner et al., 2018), which are characterized by a slow progressive loss of neurons or myelin sheaths and increasing disability and include Alzheimer’s disease (AD), Parkinson’s disease (PD), Huntington’s disease, and multiple sclerosis. Notably, patients with different types of TBI tend to have an increased risk of different neurodegenerative diseases. For example, moderate or severe TBI contributes to the development of late-onset neurodegenerative diseases, in particular AD and PD (Mendez, 2017). Chronic traumatic encephalopathy (CTE), a condition that shares features with neurodegenerative diseases, is linked to mild TBI (Fesharaki-Zadeh, 2019). Numerous random-effects meta-analysis (Perry et al., 2016), epidemiologic study (Gardner and Yaffe, 2015), and retrospective cohort study (Gardner et al., 2018) have lent support to these associations. Besides raising the risk, TBI lowers the age at onset of TBI-related neurodegenerative diseases (Mendez, 2017). Mechanically, it has previously been observed that pathological processes, including oxidative stress, neuroinflammation, excitotoxicity, proteinopathies, and mitochondrial dysfunction, may persist for months or years post-TBI, and these pathophysiological substrates serve as triggers yielding the progression of neurodegenerative diseases (Gentleman et al., 2004; Johnson et al., 2013a; Loane et al., 2014; Zhou et al., 2020a). Accordingly, targeting these post-TBI pathological processes holds the promise for the intervention of TBI and late-onset neurodegenerative diseases.

**FIGURE 1:**
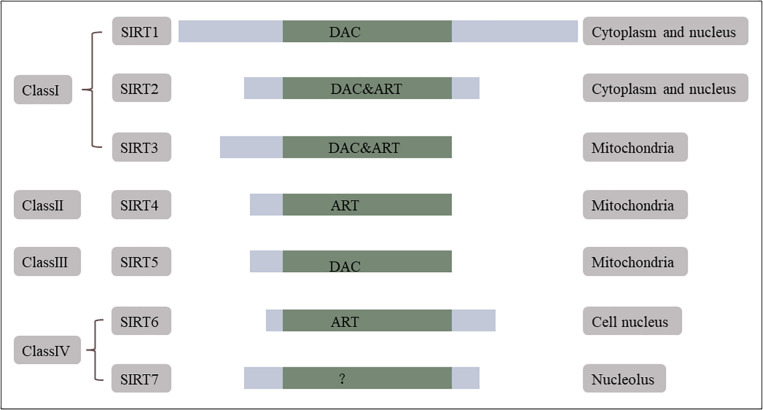
Mammalian sirtuins. Mammals have seven sirtuins. They are classified into four groups. SIRT1, 2, 3 are class I, SIRT4 is class II, SIRT5 is class III, and SIRT6, 7 are class IV. All of them have a conserved NAD^+^-dependent catalytic core domain. Each of the sirtuins has unique additional N-terminal and C-terminal sequences. SIRT1, 5 tend to show NAD^+^-dependent deacetylase (DAC) activity first whereas SIRT4, 6 are likely to express its mono-ADP-ribosyl transferase (ART) activity. Sirtuins have distributed cellular localization, mitochondria (SIRT3, 4, 5), cell nucleus (SIRT6), nucleolus (SIRT7), and both in cytoplasm and nucleus (SIRT1, 2). Reprinted and modified from *International Journal of Biological Sciences*, 7 (5), Xiaoling Li, Mammalian Sirtuins and Energy Metabolism, 575–587, 2011 (Li and Kazgan, 2011).

Homologs of Silent information regulator 2 (Sir2) are collectively known as sirtuins (Gomes et al., 2020). The mammalian sirtuin family has seven members, which are classified into four groups: class I (SIRT1–3), class II (SIRT4), class III (SIRT5), and class IV (SIRT6 and 7) (Figure 1). All sirtuins have an evolutionarily conserved nicotinamide-adenine dinucleotide (NAD^+^)-dependent catalytic core domain (Beauharnois et al., 2013; Khoury et al., 2018), and each sirtuin has unique N-terminal and C-terminal sequences (Michan and Sinclair, 2007; Li and Kazgan, 2011). SIRT1 and SIRT5 tend to elicit NAD^+^-dependent deacetylase activity, whereas SIRT4 and SIRT6 are likely to express mono-ADP-ribosyl transferase activity (Lin et al., 2012). Sirtuins occupy different cellular locations including mitochondria (SIRT3–5), the cell nucleus (SIRT6), the nucleolus (SIRT7), and between the cytoplasm and nucleus (SIRT1, 2) (Gomes et al., 2020; Zhou et al., 2020c). In addition, sirtuins are widespread in brain cells, such as neurons (Zakhary et al., 2010; Han et al., 2014; Satoh et al., 2017; Satoh and Shimokawa, 2019), astrocyte (Li et al., 2017), and microglia (Chen et al., 2015).

Sirtuins are of vital interest in oxidative stress, neuroinflammation, blood–brain barrier (BBB) permeability, astrocyte activation, and neural apoptosis in brain injuries. To date, studies regarding SIRT1 and SIRT3 have provided direct evidence about the functions of sirtuins in TBI. Despite the lack of evidence, other sirtuins may also intervene in similar pathophysiology in TBI, as have been shown in other models such as subarachnoid hemorrhage (SAH), ischemic stroke, and ischemia/reperfusion (I/R) injury. In addition, sirtuins also have some considerable impact on neurodegenerative diseases, such as AD and PD. Based on the epidemiological association and pathological similarities of TBI and neurodegenerative diseases, we propose that sirtuins may be a promising therapeutic target for TBI and related neurodegenerative diseases. In this review, we will dissect the similarity on the pathophysiological processes between TBI and neurodegenerative diseases and summarize the neuroprotective effects of sirtuins in these pathophysiological processes. We will also outline sirtuin modulators for pharmacotherapeutics and the probable difficulties in the development of pharmacological agents.

## Pathophysiology of TBI and Related Neurodegenerative Diseases

### TBI Pathophysiology

TBI is classified into mild, moderate, and severe type, which can lead to different degrees of primary and secondary brain injuries (Cornelius et al., 2013; Khatri et al., 2018). Primary injuries are attributed to the direct result of physical injuries, including hemorrhage and tissue damage (Greve and Zink, 2009). Secondary injuries include cellular hyperexcitability, vasogenic and cytotoxic edema, hypoxia–ischemia (Karve et al., 2016), microglia polarization (Chen et al., 2018a), and astrocyte activation (Li et al., 2017; Zhou et al., 2020b). Reducing primary injuries depends on preventive measures whereas second injuries are sensitive to therapeutic measures (Cornelius et al., 2013). Evidence suggests that second injuries considerably determine the outcomes of TBI patients (Cornelius et al., 2013). Therefore, in this review, we mainly focus on the second injuries and corresponding therapeutic measures.

**FIGURE 2:**
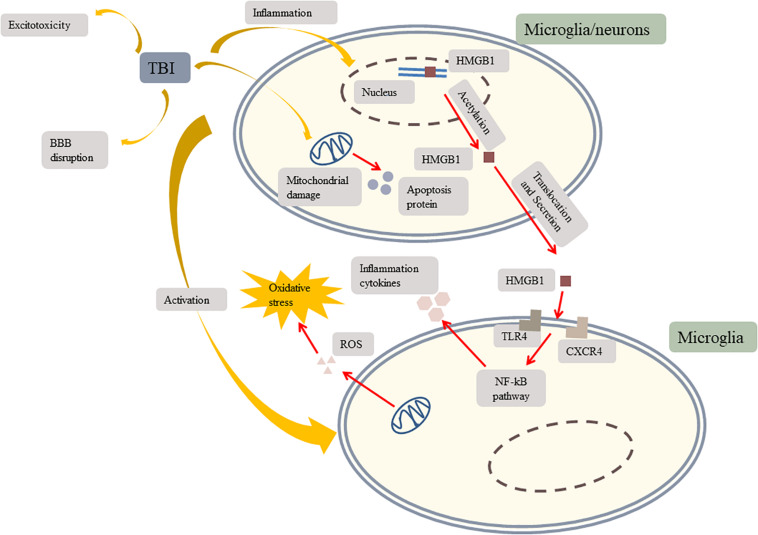
TBI induces inflammation, mitochondrial damage, microglial activation, excitotoxicity, and BBB disruption. TBI results in HMFB1 translocation and secretion. Extracellular HMGB1activates TLR4 and CXCR4 to trigger the NF-kB pathway which induces microglia secreting inflammation cytokines to further activate other microglia. TBI induces mitochondrial damage. Damaged mitochondria produce apoptosis protein and ROS to aggravate oxidative stress. Besides, TBI leads to excitotoxicity and BBB disruption which makes inflammation cytokines leakage more severe. TBI, traumatic brain injury; TLR4, toll-like receptor 4; CXCR4, chemokine (C-X-C motif) receptor 4; BBB, blood–brain barrier; HMGB1, high-mobility group box 1; NF-κB, nuclear factor-κB; ROS, reactive oxygen species.

Second injuries can further induce metabolic disorders (Hiebert et al., 2015), vascular abnormalities (Cornelius et al., 2013), extensive neuroinflammation (Toklu and Tümer, 2015; Simon et al., 2017), oxidative stress, microglial activation, and excitotoxicity (Zhou et al., 2020a). TBI-induced mitochondrial dysfunction causes cellular metabolic alterations, excitotoxicity, and endogenous antioxidant system exhaustion (Cornelius et al., 2013). The excessive production of reactive oxygen species (ROS) leads to lipid peroxidation, cytotoxicity, necrotic cell apoptosis, and oxidative stress, which in turn, exacerbate mitochondrial dysfunction (Cornelius et al., 2013; Zhou et al., 2020a). Besides, mitochondrial dysfunction can affect cell membrane permeability, resulting in the extensive release of apoptotic proteins (Chen et al., 2018b). Neuroinflammation, the physiological response to the injury, triggers the release of cytokines and chemokines and activates microglia and astrocytes (Cornelius et al., 2013). A recent study of brain injury identified a significant number of rod-shaped microglia arranged like the carriages of a train (Holloway et al., 2019). Microglia are the primary immune sentinels of the central nervous system responding to inflammatory events (Russo and McGavern, 2016) and the main source of ROS (Rangarajan et al., 2015). Studies provided evidence that microglial activity persists for a long-term period in the brain of TBI survivors (Gentleman et al., 2004; Johnson et al., 2013a; Zhou et al., 2020a). Moreover, vascular abnormalities may disrupt the BBB and cause encephaledema (Cornelius et al., 2013). Furthermore, TBI is a common cause of olfactory dysfunction, which is mediated by excitotoxicity, an intercellular cascade initiated by excessive release of glutamate and hyperactivation of the glutamatergic receptors (Marin et al., 2019; Figure 2).

### Mechanisms Underlying TBI-Induced Neurodegenerative Diseases

TBI is one of the well-established contributing factors for late-onset neurodegenerative diseases. TBI may alter the normal trajectory of the aging processes of the affected individual. Multiple injuries produce synergistic effects and accelerate neurodegeneration (DeKosky and Asken, 2017). The common causes of neurodegenerative diseases are neuroinflammation, excitotoxicity, oxidative stress, mitochondrial dysfunction, and proteinopathies, all of which are involved in the pathophysiology of TBI (Zhou et al., 2020a). Inflammation is a normal immune response coping with injuries or harmful stimuli, yet overactive inflammation damages the brain. Post-mortem and animal model data indicate that oxidative stress and neuroinflammation can persist for years after the acute phase of injury as a result of chronic microglial activation (Gentleman et al., 2004; Loane et al., 2014; Zhou et al., 2020a). According to Johnson et al.’s (2013a) research, reactive microglia were still present in 28% brains over a year after a single TBI. Excitotoxicity refers to neuronal cell death caused by the toxic action of excitatory amino acids (Dong et al., 2009). Glutamate is the primary excitatory amino acid neurotransmitter in the central nervous system and is associated with neuronal degeneration, aging, and death (Hernández et al., 2018). Oxidative stress triggers excessive free radical production, leading to neuronal death and stimulates inflammation (Khatri et al., 2018). TBI-induced mitochondrial dysfunction decreases the metabolic energy supply and interferes with synaptic function (Goldstein et al., 2012). The resulting depletion of energy stores stimulates the release of ROS, which in turn, aggravates oxidative stress. Besides, Daglas and Adlard (2018) indicated that the detrimental role of iron deposits in the TBI pathogenesis may constitute one of the reasons for boosting late-onset neurodegenerative diseases.

AD, one of the most common neurodegenerative diseases, is characterized by the aggregation of β-amyloid protein and tau protein. PD, which is caused by the selective degeneration of dopamine and is characterized by bradykinesia and rigidity, is the second most common neurodegenerative disease (Shi et al., 2017). Studies showed that TBI is one of the strong environmental contributing factors for AD and PD (Hayes et al., 2017; Al-Dahhak et al., 2018; Gardner et al., 2018). One of the possible reasons is that TBI causes diffuse axonal injury and leads to proteinopathies. TBI causes mechanical trauma to axons and induces the excessive release of glutamate, which may substantially alter neuronal metabolism (Giza and Hovda, 2014). Dysregulated axonal transport systems lead to axonal swelling, which may disrupt the delivery of necessary proteins and ultimately lead to focal nerve disconnection (Johnson et al., 2012, 2013b). In damaged axons, accumulated amyloid precursor proteins and amyloid β-peptide are markers of axon injury (Smith et al., 2003). These markers are frequently found existing in the brains of TBI survivors (Ikonomovic et al., 2004) and post-mortem brains (Smith et al., 2003). Although the mechanism underlying TBI-induced PD is not clear, it may involve mitochondrial dysfunction and oxidative stress (Shi et al., 2017). Amyloid β-peptide accelerates tau protein pathology, but the underlying mechanism remains unknown (Yin et al., 2018). TBI causes hyperphosphorylation, misfolding, and aggregation of tau proteins, which then form neurofibrillary tangles (NFTs) (Johnson et al., 2012; Du et al., 2014). Hyperphosphorylated tau proteins are released into the extracellular space and stimulate microglia and astrocytes to produce pro-inflammatory cytokines (e.g., IL-1β and TNFα), which further activate tau kinases and aggravate tau hyperphosphorylation (Fesharaki-Zadeh, 2019). Besides phosphorylation, tau acetylation at Lys174 is a crucial change during the post-translational modification of tau proteins, determining the toxicity in mice, tau homeostasis, and the initial step of AD (Min et al., 2015).

CTE, a neurodegenerative disease caused by repeated mild or concussive head injuries, is characterized by an abnormal accumulation of hyperphosphorylated tau protein (Johnson et al., 2012). Clinically, patients with CTE present with two distinct phenotypes: affective changes and cognitive impairment (Fesharaki-Zadeh, 2019). Several large epidemiological studies suggest that a single severe TBI is a significant risk factor for the later development of AD (Johnson et al., 2017). The effects of TBI are long-term and extend beyond the immediate acute phase. Previous findings suggest that neuronal densities in the hippocampus and thalamus continue to decrease 1 year or more after a severe TBI (Maxwell et al., 2003, 2006).

Taken collectively, it appears likely that targeting the aforementioned pathological processes post-TBI holds the promise for the treatment of TBI as well as the treatment and even prevention of TBI-induced neurodegenerative diseases.

## Potential Roles of Sirtuins in TBI and Related Neurodegeneration

After TBI, the expressions of sirtuins increase (Yang et al., 2017). Previous research has established that sirtuins exert a neuroprotective effect after TBI (Zhao et al., 2012; Yuan et al., 2016; Chen et al., 2017). Nevertheless, related research concerning the functions of sirtuins, particularly SIRT2, 4, 5, 6, 7, in TBI remains insufficient. Notably, sirtuins are also reported to be involved in the pathophysiology of other acute brain injury models, such as SAH, ischemic stroke, and I/R injury, whose pathophysiologies have some similarities with that of TBI (Table 1). Altogether, sirtuins regulate inflammation, oxidative stress, astrocyte activation, BBB permeability, axon elongation, and synaptic plasticity during acute brain injuries, all of which are characteristic pathological features of TBI (Fujita and Yamashita, 2018). Furthermore, some researchers indicated that sirtuins could reduce the accumulation of amyloid β peptide (Ramesh et al., 2018) and tau protein (Du et al., 2014), suggesting their preventive potential for TBI-induced neurodegenerative diseases. In this section, we outline the potential underlying mechanisms of sirtuins in TBI and related neurodegeneration, particularly with regards to AD. Of note, some of these candidate mechanisms are deduced from the models of other acute brain injuries, which are highlighted in corresponding sentences.

**TABLE 1:** Function of sirtuins in brain injury.

Sirtuins	Roles	Pathway/Mechanism	Models	Activation/Overexpression	Inhibition/Silence
SIRT1	Protective	Astrocyte activation	TBI	**↓** (Li et al., 2017)	–
	Protective	BBB integrality	SAH and aging	↑ (Qian et al., 2017; Zhao et al., 2017; Stamatovic et al., 2019)	↓ (Zhou et al., 2014; Zhao et al., 2017; Stamatovic et al., 2019)
	Protective	Inflammation	TBI	↓ (Zou et al., 2018)	–
	Protective	Microglia activation and polarization	TBI	↓ (Chen et al., 2017, Chen et al., 2018a)	–
	Protective	Oxidative stress	TBI	↓ (Zou et al., 2018)	–
	Protective	Excitotoxic lesion	Cortical neurons	↓ (Jia et al., 2016; Marin et al., 2019)	–
	Protective	Vascular repair	Ischemic brain tissues	↑ (Choi et al., 2017)	–
	Protective	Endothelial cell apoptosis	SAH	–	↑ (Zhou et al., 2014)
	Protective	Apoptosis of neurons	TBI	↑ (Zhao et al., 2012)	↓ (Zhao et al., 2012)
	Protective	Axon genesis and axon elongation	Embryonic hippocampal neurons	↑ (Li et al., 2013)	–
	Protective	Mitochondrial damage	TBI	↓ (Yang et al., 2017)	↑ (Yang et al., 2017)
	Protective	Tau hyperphosphorylation	Rat model of brain insulin resistance	↓ (Du et al., 2014)	
SIRT2	Protective	BBB integrality	TBI	–	↓ (Yuan et al., 2016)
	Protective	Microglia and inflammation	LPS induced neuroinflammation models	↓ (Pais et al., 2013)	–
SIRT3	Detrimental	Ceramide accumulation	Stoke	↑ (Novgorodov et al., 2016)	–
	Protective	Oxidative stress	TBI; SAH; I/R; OGD	↓ (Rangarajan et al., 2015; Shi et al., 2017; Zhao et al., 2018; Tu et al., 2019)	↑ (Rangarajan et al., 2015; Huang et al., 2016; Shi et al., 2017; Tu et al., 2019)
	Protective	Autophagy of neurons	OGD	↑ (Dai et al., 2017)	–
	Protective	Excitotoxicity	Cortical neurons	↓ (Kim et al., 2011)	–
	Protective	Amyloid β peptide	Chinese Hamster Ovarian (CHO) cells	↓ (Ramesh et al., 2018)	–
SIRT4	Protective	Glutamate transport function	Sirt4 KO mice	–	↓ (Shih et al., 2014)
	Protective	Excitotoxicity	Cortical neurons	↓ (Marin et al., 2019)	–
SIRT5	Detrimental	BBB permeability	I/R	–	↓ (Diaz-Cañestro et al., 2018)
SIRT6	Protective	Oxidative stress	Stroke; I/R; OGD	↓ (Zhang W. et al., 2017; Liberale et al., 2019)	↑ (Liberale et al., 2019)
	Protective	BBB integrality	Stoke	↑ (Liberale et al., 2019)	↓ (Liberale et al., 2019)
SIRT7	Protective	Oxidative stress	I/R	↓ (Lv et al., 2017)	–
	Protective	Apoptosis	I/R	↓ (Lv et al., 2017)	–

### Sirtuins Reduce Inflammation

In TBI models, existing research has shown the critical role played by SIRT1 to reduce neuroinflammation (Chen et al., 2017). Moreover, SIRT2 was observed to alleviate inflammation in LPS-induced neuroinflammation models (Pais et al., 2013).

SIRT1 reduces inflammation by modulating high mobility group box 1 (HMGB1). SIRT1 maintains HMGB1 in a deacetylated state to control neuroinflammation resulting from microglial activation after TBI. Acetylated HMGB1 is required for HMGB1 transcription and extracellular secretion. HMGB1 is a non-histone-binding protein. Under normal conditions, HMGB1 binds to DNA and regulates transcription and translation in the nucleus. After TBI damage, HMGB1 translocates from the nucleus to the extracellular space. Extracellular HMGB1 induces microglial activation, which leads to further HMGB1 translocation (Chen et al., 2017). Extracellular HMGB1 binds to receptors on the cell surface, including toll-like receptor 4 and chemokine receptor 4 (Wang et al., 2017). HMGB1 activates receptors via medullary differentiation factor (MyD88) and non-MyD88-dependent pathways, triggering a signaling cascade in the microglia via the nuclear factor-κB (NF-κB) pathway. Nuclear translocation of NF-κB induces microglial polarization and cascade amplification of inflammatory cytokines (Chen et al., 2018a). Furthermore, SIRT1 inhibits activation of the NOD-like receptor (NLR) family pyrin domain containing 3 (NLRP3) inflammasome to attenuate inflammation after TBI. NLRP3 serves as a platform to produce interleukin-1β (IL-1β), an inflammatory initiating cytokine in a caspase reaction (Zou et al., 2018). Inflammation flooding further activates microglia, which in turn, aggravates inflammation (Chen et al., 2017). Previous studies have shown that microglial activation up to 1 year after TBI may cause chronic inflammation (Gentleman et al., 2004; Loane et al., 2014). Activated microglia have been identified around lesions in various neurodegenerative diseases (Xu et al., 2016). Excessive activation of microglia further damages neurons by releasing cytotoxic factors and accelerating the progression of neurodegenerative diseases (Glass et al., 2010). Moreover, SIRT1 was shown to mitigate astrocyte activation by inhibiting the p38 mitogen-activated protein kinase (MAPK) signaling pathway in an experimental model of TBI (Li et al., 2017). Astrocyte activation results in the overproduction of glial scar and inflammatory cytokines, which further activates the astrocytes, creating a positive inflammatory feedback loop that may injure the remaining neurons (Gayen et al., 2020).

### Sirtuins Reduce Excitotoxicity

Excitotoxicity is implicated in the pathogenesis of several neurodegenerative diseases. Excessive release of glutamate results in glutamate receptor hyperactivation, calcium buffering impairment, free radical overproduction, and mitochondrial hyperpermeability, consequently leading to further neuronal degeneration and programmed cell death (Dong et al., 2009). Studies have shown that SIRT1, SIRT3, and SIRT4 are key players in reducing excitotoxic lesion in cortical neurons models (Kim et al., 2011; Jia et al., 2016; Marin et al., 2019).

An *in vitro* study in cortical neurons found that SIRT1 deacetylated and decreased peroxisome proliferator-activated receptor-coactivator 1α (PGC1α) to reduce glutamate excitotoxicity (Jia et al., 2016). PGC1α is a key factor in the maintenance of mitochondrial biogenesis and respiratory function (Scarpulla, 2011). Previous studies showed that SIRT4 promotes glutamate transport capacity by inhibiting excitotoxicity (Shih et al., 2014) and interacts with glutamine catabolism by hampering glutamate dehydrogenase (GDH) (Marin et al., 2019). Glutamine catabolism is crucial during the DNA repair response (Jia et al., 2016). Excessive glutamine catabolism leads to neuronal dysfunction and cell death (Jia et al., 2016). Furthermore, mitochondrial SIRT3 expression increases after poly(ADP-ribose) polymerase-1-mediated NAD depletion in cortical neurons. Kim et al. (2011) concluded that SIRT3 is essential for neuroprotection against excitotoxicity. The authors showed that NAD depletion further increased ROS production, aggravated oxidative stress, and upregulated the expression of SIRT3 in mitochondria.

### Sirtuins Improve Mitochondrial Function and Reduce Oxidative Stress

Mitochondrial dysfunction is a fundamental pathogenic event in most neurodegenerative diseases. Findings from rat models of TBI have revealed that ceramide is the first lipid to accumulate in the mitochondria after TBI (Roux et al., 2016), suggesting it may serve as a therapeutic indicator (Barbacci et al., 2017). Ceramide accumulation causes mitochondrial dysfunction (Novgorodov et al., 2016), which is associated with increased mitochondrial fission and excessive ROS production (Zhao et al., 2018).

According to existing research, SIRT1 and SIRT3 have been detected to alleviate oxidative stress in TBI models (Rangarajan et al., 2015; Zou et al., 2018). The study by Yang et al. (2017) showed that SIRT1 reduces the mitochondrial damage to play its neuroprotective roles in TBI. SIRT3, SIRT6, and SIRT7 showed their functions of reducing oxidative stress in other brain models (Lv et al., 2017; Shi et al., 2017; Zhang W. et al., 2017; Zhao et al., 2018; Liberale et al., 2019; Tu et al., 2019).

SIRT1 has been shown to improve mitochondrial function by inhibiting the p38 MAPK pathway in animal models of TBI. Given that MAPK protein is associated with multiple phosphorylation/dephosphorylation signaling cascades, SIRT1 may inhibit this pathway by dephosphorylating p38 MAPK (Yang et al., 2017). A previous study found that SIRT1 activity was downregulated in the brains of patients with neurodegenerative diseases including PD. Furthermore, the authors reported that SIRT1 reduced oxidative stress-induced neural cell death and concluded that SIRT1 is a pro-survival protein that is downregulated under cellular stress (Singh et al., 2017). These findings suggest that SIRT1 has the potential to reduce oxidative stress in TBI and associated neurodegenerative diseases.

Investigations of the effects of SIRT3 on mitochondrial dysfunction and oxidative stress have yielded contradictory findings. Results from animal models provide evidence supporting a promoting role for SIRT3 in the accumulation of ceramide within the mitochondria (Novgorodov et al., 2016), which serves as a causal factor of mitochondrial dysfunction by inhibiting the activity of respiratory chain complex III (Gudz et al., 1997; Anderson and Sims, 1999; Yu et al., 2007). Indeed, SIRT3 deacetylates ceramide synthase 1, 2, and 6 to keep them in hyperacetylated state (primed state), propelling the ceramide accumulation (Novgorodov et al., 2016). However, some study showed that the inhibition of SIRT3 had no beneficial effects on brain injury in a model of stroke (Verma et al., 2019). Inversely, inhibition of SIRT3 in microglia leads to excessive ROS production. SIRT3 deacetylates Forkhead box O 3a (Foxo3a), a transcription factor that transactivates antioxidant genes, to enable it to translocate into the nucleus. Thus, SIRT3 increases the production of antioxidants, including catalase and manganese superoxide dismutase (mnSOD), to protect against oxidative stress (Rangarajan et al., 2015). Another study pointed out that it is a compensatory increase in SIRT1 rather than the inhibition of SIRT3 that exerts neuroprotective effect (Verma et al., 2019). Not only that, recent work by researchers proved that SIRT3 exerted neuroprotective effect against oxidative stress not only in models of TBI (Rangarajan et al., 2015) but also in SAH (Huang et al., 2016), I/R (Zhao et al., 2018; Tu et al., 2019), and oxygen-glucose deprivation (OGD) (Wang et al., 2015) models. Moreover, SIRT3 protects dopaminergic neurons against oxidative stress (Shi et al., 2017). The degeneration of dopaminergic neurons is the primary cause of PD. SIRT3 regulation of mnSOD activity via deacetylation at the lysine 68 site attenuates oxidative stress and restores mitochondrial membrane potential in dopaminergic neurons (Shi et al., 2017). Collectively, these findings support SIRT3-induced attenuation of oxidative stress after TBI and related neurodegeneration.

SIRT6 locates in the nucleus, where it promotes resistance to DNA damage and oxidative stress by supporting DNA double-strand break repair (Beauharnois et al., 2013). SIRT6 levels decrease after I/R injury. Nuclear factor erythroid-derived 2-like 2 (NRF2) is activated via the deacetylation by SIRT6. NRF2 is a basic leucine zipper transcription factor that regulates the expression of antioxidant proteins, such as heme oxygenase-1 and SOD, that resist oxidative stress and have a neuroprotective function (Zhang W. et al., 2017). Although SIRT7 is expressed ubiquitously in the cortex, striatum, hippocampus, and thalamus (Islam et al., 2018), its function in the brain is unclear. A recent study in an I/R model found that SIRT7 regulated apoptosis and resisted oxidative stress via deacetylation of the p53 protein (Lv et al., 2017). This finding suggests that SIRT6 and 7 may have similar effects on oxidative stress after TBI.

### Sirtuins Mitigate Proteinopathies

Sirtuins are a kind of longevity assurance factor and involved in the promotion of healthy aging mechanisms (Braidy et al., 2012). Sirtuins have some special functions on lowering the risk of neurodegenerative diseases such as reducing the accumulation of tau proteins and amyloid β-peptide (Braidy et al., 2012; Du et al., 2014).

Tau proteins and amyloid β-peptide accumulations serve as indispensable parts in the development of AD (Braidy et al., 2012). Cytological studies have shown that SIRT1 in growth cones promotes axon genesis and elongation in embryonic hippocampal neurons (Li et al., 2013). The accumulation of amyloid precursor protein and amyloid β-peptide in injured axons after TBI has been demonstrated *in vivo* (Smith et al., 2003) and *in vitro* (Tang et al., 2017) studies. These pathologies are associated with AD (Smith et al., 2003). SIRT1 locates in the distal regions of the axon, the axonal growth cone in particular. SIRT1 regulates the deacetylation and activation of protein kinase B (Akt). Akt is the upstream inhibitory kinase of glycogen synthase kinase3 (GSK3). Akt activation inhibits GSK3 activity (Li et al., 2013). GSK3 is a multifunctional serine/threonine kinase and a key regulator of neurogenesis, polarization, neurite outgrowth, and plasticity in the nervous system (Hur and Zhou, 2010). Intracellular mechanisms involved in axon genesis include actin filaments and the reorganization of microtubules (Li et al., 2013). In addition to reducing amyloid β-peptide accumulation, SIRT1 upregulates the amount of lysosome in primary astrocytes to promote the degradation of amyloid β-peptide by its deacetylase activity (Li M.Z. et al., 2018). Recent studies showed that SIRT1 may exert its anti-amyloidogenic effects by direct activating gene *ADAM10*, which encodes disintegrin protein (Braidy et al., 2012). Furthermore, an *in vitro* study in Chinese hamster ovarian cells found that SIRT3 reduced ROS production and lipid peroxidation and improved mitochondrial function, resulting in the reduction of amyloid β-peptide production (Ramesh et al., 2018).

Hyperphosphorylated tau protein accumulation is widespread after a single TBI (Johnson et al., 2012). Hyperphosphorylated tau proteins misfold and aggregate to form NFTs, consequently resulting in impaired tau function (Johnson et al., 2012). Hyperphosphorylated tau proteins are liberated into the extracellular space, stimulating microglia and astrocytes to release pro-inflammatory cytokines (e.g., IL-1β and TNFα), which further activate tau kinases and aggravate tau hyperphosphorylation (Fesharaki-Zadeh, 2019). In addition to phosphorylation, acetylation is a crucial step in the post-translational modification of tau proteins. The acetylation of tau protein has been demonstrated in all stages of AD. An *in vitro* study in cortical neurons argued that SIRT3 deacetylated tau proteins, which attenuated the pathological accumulation of tau proteins (Yin et al., 2018). Du et al.’s (2014) research has shown that SIRT1 suppressed tau protein accumulation in hippocampus neurons in a rat model of brain insulin resistance.

### Sirtuins and BBB Integrity

The BBB is an interface between the brain parenchyma and cerebral circulation. BBB permeability is regulated by pericytes, astrocytes, and cerebral microvascular endothelial cells through the expression of tight and adherent junctions (Liberale et al., 2019). BBB disruption serves as one of the critical parts in the development of AD and other neurodegenerative disorders. Disruption of the BBB allows neurotoxic cells, pathogens, and inflammatory cytokines to leak from the cerebral circulation to the brain parenchyma, where they activate inflammatory and immune responses associated with severe TBI and the development of neurodegenerative diseases (Qian et al., 2017; Sweeney et al., 2018; Zhou et al., 2020a).

SIRT2 serves as a pivotal factor in BBB integrity. SIRT2 inhibition has been shown to increase BBB permeability and exacerbate inflammation and brain edema after TBI (Yuan et al., 2016). Matrix metalloproteinase (MMP)-9 degrades tight junction proteins of BBB. SIRT2 inhibition stimulates MMP-9 expression and disturbs the integrity of BBB, allowing plasma components (e.g., macrophage and inflammatory factors) crossing the BBB more easily, consequently resulting in the exacerbation of neuroinflammation. Moreover, SIRT2 inhibition promotes microglia and macrophage activation, which intensifies the inflammatory processes. SIRT2 inhibition promotes nuclear translocation of NF-kB p65 via acetylation of the p65 subunit to increase NF-kB activity and upregulate its targets including aquaporin 4, MMP-9, and proinflammatory cytokines (Yuan et al., 2016). By contrast, SIRT5 adversely affects the maintenance of BBB integrity. SIRT5 has previously been observed to exacerbate inflammation in an experimental model of I/R injury, while SIRT5 inhibition decreased endothelial permeability of the BBB and increased the expression of occludin and claudin-5 via the phosphatidylinositol 3-kinase/Akt pathway (Diaz-Cañestro et al., 2018).

In experimental models of SAH and aging, SIRT1 prevents BBB hyperpermeability (Qian et al., 2017; Stamatovic et al., 2019). This effect can be reversed by inhibiting SIRT1 (Zhou et al., 2014). Tight junction proteins, such as claudin-5 and occludin, are the main components of the BBB structure (Kniesel and Wolburg, 2000). SIRT1 upregulates tight junction protein expression in response to brain edema to protect BBB integrity. In addition, SIRT1 attenuates damage caused by brain edema via the deacetylation of p53. P53 is a protein upregulating endothelial MMP-9 via the NF-kB pathway to reduce occlusion in animal models of SAH (Yan et al., 2008; Qian et al., 2017). Furthermore, a study of ischemic brain tissue from mice found that SIRT1 was involved in the repair of BBB vascular destruction by upregulating vascular endothelial growth factor expression in astrocytes (Choi et al., 2017). SIRT1 was proved to accelerate endothelial cell apoptosis in an animal model of SAH (Zhou et al., 2014). Furthermore, a recent study suggested that endothelial SIRT6 protected BBB integrity after an I/R injury in a stroke model; however, the mechanism is not fully understood (Liberale et al., 2019).

### Sirtuins and Neuronal Apoptosis

Sirtuins reduce the apoptosis of normal neurons and promote apoptosis in damaged neurons. TBI induces SIRT1 expression and activates the MAPK/extracellular signal-related kinase (ERK) pathway. Inhibition of SIRT1 and the MAPK/ERK pathway decreases neuronal apoptosis. Moreover, inhibition of the MAPK/ERK pathway further decreases SIRT1 levels. The dynamic relationship between SIRT1 and apoptosis has been demonstrated in *in vivo* and *in vitro* models of TBI (Zhao et al., 2012). Moreover, sirtuins play an essential role in reducing apoptosis in other models of brain injury. SIRT1 is a nicotinamide-adenine dinucleotide-dependent p53 deacetylase. P53 induces apoptosis in the acetylated state (Sykes et al., 2006). SIRT1 promotes p53 proteolysis via deacetylation (Qian et al., 2017) to inhibit p53 transactivation activity and suppress apoptosis in response to oxidative stress in an experimental model of SAH. SIRT3 was found to promote autophagy of damaged neurons to protect ischemic neurons in a model of OGD (Dai et al., 2017). Cortical neurons overexpress SIRT3 under ischemic and hypoxia conditions, further increasing the phosphorylation of adenosine 5′-monophosphate (AMP)-activated protein kinase (AMPK), which inhibits the phosphorylation of mTOR and induces autophagy of ischemia neurons (Dai et al., 2017).

## Sirtuins as Promising Therapeutic Targets

Given that primary TBIs are irreversible, interventional strategies to prevent secondary injuries and promote plasticity and the recovery process are of primary concern. As mentioned previously, the sirtuin family are vividly involved in several TBI-induced pathophysiological processes including microglial and astrocyte activation, inflammation, proteinopathies, oxidative stress, and mitochondrial damage, all of which may facilitate the onset of neurodegenerative diseases. Moreover, sirtuins have a wide range of effects on BBB integrity, apoptosis, and excitotoxicity in other *in vivo* and *in vitro* models of brain injury. As a result of these pleiotropic effects, sirtuins have been acknowledged to be emerging targets for TBI and TBI-related neurodegeneration (Donmez and Outeiro, 2013; Foolad et al., 2019).

There is a growing body of literature that recognizes the therapeutic potential of the modulators of sirtuins (Table 2). Although these modulators have not been tested clinically, there is an abundance of preclinical data in cortical neuron cultures and animal models that describe specific mechanisms of modulator actions. Identification of a modulator that allows precise control of sirtuin expression will enable clinicians to treat TBI and prevent pathological processes that trigger late-onset neurodegenerative diseases.

**TABLE 2:** Modulators of sirtuins.

Sirtuins	Modulators	Pathways/Mechanism	Functions	References
SIRT1	Resveratrol	AMPK/SIRT1/autophagy	↓ Autophagy	Wan et al., 2016; Li and Han, 2018
		BDNF/trkb	↑ Recovery of motor function	Shi et al., 2016
		Camp/Bcl-2	↓ Apoptosis	Shin et al., 2012
		NLRP3	↓ Oxidative stress and I/R injury	He et al., 2017; Zou et al., 2018
	Rolipram (Phosphodiesterase-4 inhibitor)	NF-kB	↓ Neuroinflammation and neuronal loss	Peng et al., 2018
		**Akt (Protein kinase B)**	↓ Neuronal apoptosis	Li Q. et al., 2018
	Omega-3	Autophagy pathway	↓ Neuronal apoptosis and microglia polarization	Chen et al., 2018b
	Melatonin	Melatonin receptor/Sirt1/NF-kb	↓ Apoptosis, neuroinflammation, mitochondrial dysfunction and oxidative stress	Yang et al., 2015; Zhao et al., 2017
	Astaxanthin	TLR4	↓ Inflammation	Zhang et al., 2019
	Dexmedetomidine	NFAT5/SIRT1/Nrf2	↓ I/R injury	Chen et al., 2019
	Agomelatine	NF-kb/p65	↓ Inflammation, oxidative stress and neuronal apoptosis	Savran et al., 2020
	Saponin	Akt/SIRT1/FOXO3a/PGC-1*α*	↓ Cell apoptosis and mitochondrial dysfunction	Duan et al., 2019
	Estrogen	AMPK	↓ Ischemic brain injury	Guo et al., 2017
	Icariin	PGC-1alpha	↓ Ischemic brain injury	Zhu et al., 2010
	Ginsenoside	NF-KB	↓ Inflammation	Cheng et al., 2019
	Salidroside	SIRT1/FOXO3α	↓ I/R injury	Xu et al., 2018
	Arctigenin	NLRP3	↓ Inflammation	Zhang W. et al., 2017
	Alpha-lipoic acid	PGC-1α	↓ Oxidative stress	Fu et al., 2014
	Lithospermate B	NF-KB	↓ Neuroinflammation and apoptosis	Lv et al., 2015; Peng et al., 2018
	Nampt	NAD+/Sirt1/AMPK	↓ Neuron apoptosis	Wang et al., 2011
		Mtor/S6K1	↑ Autophagy	Wang et al., 2012
	Curcumin	Not mentioned	↓ I/R injury	Miao et al., 2016
	Oxymatrine		↓ Autophagy	Zhou et al., 2018
	Magnolol		↓ Inflammation and apoptosis	Kou et al., 2017
	Liraglutide		↑ Mitochondrial involved brain repair	He et al., 2020
	Vitamin E		↓ Oxidative stress	Aiguo et al., 2010
SIRT2	DHA	Not mentioned	↓ Oxidative stress	Wu et al., 2011
	Hydrogen-rich saline		↓ Oxidative stress	Hou et al., 2012
SIRT3	Minocycline	Phd-2	↑ BBB integrity	Yang et al., 2015
	Melatonin	NLRP3	↓ ROS production	Yang et al., 2018
	Magnolol	Not mentioned	↓ Oxidative stress and amyloid proteinβ	Ramesh et al., 2018
	LMWF		↓ Oxidative stress and mitochondrial disfunction	Wang et al., 2016
SIRT6	Bexarotene	PPARγ/foxo3a	↓ Neuroinflammation	Zuo et al., 2019

Resveratrol (3, 5, 4′-trihydroxystilbene) is one of the most effective natural SIRT1-activating compounds. Resveratrol is a natural polyphenolic substance extracted from grapes that has been found to increase SIRT1 activity about 10-fold. By modifying at the B ring 4′ position, scientists developed a variety of synthetic resveratrol derivatives to lower toxicity to cells or improve potency (Dai et al., 2018). Resveratrol and its derivatives play various roles in apoptosis (Shin et al., 2012), autophagy (Wan et al., 2016; Li and Han, 2018), oxidative stress (He et al., 2017), and recovery of motor functions (Shi et al., 2016). Moreover, resveratrol modulates the activity of SIRT3 and SIRT5 (Dai et al., 2018). Several other natural extracts also activate SIRT1, including magnolol (Kou et al., 2017), oxymatrine (Zhou et al., 2018), curcumin (Miao et al., 2016), salvianolic acid B (Lv et al., 2015), arctigenin (Zhang S. et al., 2017), astaxanthin (Zhang et al., 2019), salidroside (Xu et al., 2018), ginsenoside (Cheng et al., 2019), icariin (Zhu et al., 2010), low molecular weight fucoidan (Wang et al., 2016), and saponin from *Aralia taibaiensis* (Duan et al., 2019). These findings suggest that Chinese traditional medicine may be a source of sirtuin modulators.

Moreover, some hormones modulate SIRT1. Melatonin, a kind of hormone mainly secreted by the pineal gland, regulates circadian rhythms. Melatonin is a powerful antioxidant that attenuates early brain injury–induced oxidative stress and neuronal apoptosis by activating the SIRT1/NF-kB pathway (Zhao et al., 2017). Furthermore, melatonin upregulates the expression of SIRT3 to reduce the production of ROS and resist oxidative stress (Yang et al., 2018). Estrogen also regulates SIRT1. Estrogen restores resistance to cell apoptosis and attenuates ischemic brain injury via the SIRT1/AMPK pathway (Guo et al., 2017). Activation of the SIRT1/AMPK pathway may underlie the neuroprotective effect of estrogen.

Some drugs that are not originally developed to regulate sirtuins have currently been found to have neuroprotective properties via a SIRT-involved pathway. For instance, the long-acting glucagon-like peptide-1 analogs, liraglutide (He et al., 2020), agomelatine (Savran et al., 2020), and dexmedetomidine (Chen et al., 2019), exert a SIRT1-dependent neuroprotective effect and critically regulate the metabolism. Furthermore, the antibiotic, minocycline, modulates SIRT3 to protect BBB integrity (Yang et al., 2015), and bexarotene attenuates neuroinflammation by modulating SIRT6 (Zuo et al., 2019).

Intermittent fasting, and daily caloric (Ran et al., 2015) or protein restrictions (de Carvalho et al., 2019) have been shown to reduce inflammation and oxidative stress by upregulating SIRT1. Specifically, reduced energy intake without affecting nutritional requirements has a neuroprotective effect after brain injury (Ran et al., 2015). Besides, docosahexaenoic acid (Wu et al., 2011), vitamin E (Aiguo et al., 2010), and hydrogen-rich saline (Hou et al., 2012) reduce oxidative stress. The antioxidant, alpha-lipoic acid, reduces oxidative stress by upregulating SIRT1 (Fu et al., 2014).

Besides, NAD^+^ nicotinamide mononucleotide (NMN; precursor of NAD^+^) provides neuroprotection by directly activating sirtuins (Klimova et al., 2019). Dysregulation of NAD^+^ metabolism may cause neurodegenerative diseases and acute brain injury. NMN is a multi-targeted modulator: it activates sirtuins, inhibits mitochondrial fission, and is an NAD+ supplement. Therefore, NMN is a promising therapeutic agent for TBI and associated neurodegenerative diseases (Klimova and Kristian, 2019). NMN is an endogenous cellular metabolic compound that may have lower toxicity and fewer side effects at high doses (Park et al., 2016).

Furthermore, some enzymes modulate sirtuin. Nicotinamide phosphoribosyltransferase (Nampt) is the rate-limiting enzyme in mammalian NAD^+^ biosynthesis (Wang et al., 2011). Nampt, which is located primarily in neurons, regulates autophagy and apoptosis (Wang et al., 2012). Rolipram, a phosphodiesterase-4 inhibitor, modulates inflammation and neuronal apoptosis via different SIRT1-dependent pathways (Li Q. et al., 2018; Peng et al., 2018).

However, a better understanding of sirtuins and their molecular structures is still needed to facilitate the discovery of additional modulators. Referring to Table 1, we can find that sirtuins seem to play contradictory roles under certain circumstances, which may bring difficulties to the development of TBI pharmacological agents (Gomes et al., 2019).

## Conclusion and Limitations

TBI is an accepted risk factor for neurodegenerative diseases. The key pathological features of TBI and neurodegenerative diseases include neuroinflammation, oxidative stress, excitotoxicity, proteinopathies, and mitochondrial dysfunction, with many similarities and overlaps. Therefore, targeting these processes has the therapeutic potential for TBI and associated neurodegenerative diseases. The sirtuin family have seven members, namely SIRT1–7 and are strictly implicated in the pathological mechanisms of acute brain injuries. Among these sirtuins, SIRT1 and SIRT3 have been demonstrated to reduce the post-TBI damage by alleviating inflammation, oxidative stress and proteinopathies, neuronal apoptosis, and BBB compromise. The rest sirtuins intervene in similar pathophysiology in other brain injury models and merit further exploration in TBI models. Recent progress on sirtuin modulators has made sirtuins promising therapeutic targets. However, sirtuins may show contradictory effects under certain conditions, and certain off-target effects should be taken into consideration.

This review has some limitations. First, studies about sirtuins’ effects on TBI are not enough yet, especially SIRT2, 4, 5, 6, and 7, which lack support from direct evidence in TBI models. We speculate that functions of SIRT2, 4, 5, 6, and 7 in other kinds of brain injury models may be also valid in TBI models. Second, the demonstration of the functions of sirtuins in this article is all based on animal test results. Hence, further exploration of human and clinical evidence is warranted. Third, the hypothetical linking between TBI and neurodegenerative diseases is supported by similarities of pathological processes. We should explore more supporting evidence to offer this association a more solid foundation.

## Author Contributions

YZ and AS conceptualized the research project. QY, YZ, YuS, and YL wrote the manuscript and made the original figures. AS, YeS, and QY critically revised the texts and figures. AS, YeS, and YZ supervised the research and led the discussion. All authors contributed to the article and approved the submitted version.

## Conflict of Interest

The authors declare that the research was conducted in the absence of any commercial or financial relationships that could be construed as a potential conflict of interest.

## Abbreviations

AD: Alzheimer’s disease
Akt: protein kinase B
AMPK: adenosine monophosphate activated protein kinase
AQP4: aquaporin 4
BBB: blood–brain barrier
Cat: catalase
COX-1: cyclooxygenase-1
CTE: chronic traumatic encephalopathy
Foxo3a: forkhead box O 3a
GDH: glutamate dehydrogenase
GSK3: glycogen synthase kinase3
HMGB1: high-mobility group box 1
HO-1: heme oxygenase-1
I/R: ischemia/reperfusion
LPS: lipopolysaccharide
MAPK: mitogen-activated protein kinases
MARK: mitogen-activated protein kinase
MMP-9: matrix metalloproteinase 9
mnSod: manganese superoxide dismutase
MyD88: medullary differentiation factor
Nampt: nicotinamide phosphoribosyl-transferase
NF- κ B: nuclear factor- κ B
NFTs: neurofibrillary tangles
NLRP3: NLR family pyrin domain containing 3
NMN: NAD^+^ nicotinamide mononucleotide
NRF2: nuclear factor (erythroid-derived 2)-like 2
OGD: oxygen-glucose deprivation
PARP-1: poly (ADP-ribose) polymerase-1
PD: Parkinson’s disease
PGC1 α: peroxisome proliferator-activated receptor-coactivator 1 α
PI3K: phosphatidylinositol 3-kinase
ROS: reactive oxygen species
Sir2: Silence information regulator 2
SIRT: sirtuin
SOD: superoxide dismutase
STACs: sirtuin activating compounds
TBI: traumatic brain injury
TLR4: toll-like receptor 4.
